# Examining External Validity in Efficacy and Secondary Articles of Home-Based Depression Care Management Interventions for Older Adults

**DOI:** 10.5888/pcd9.120110

**Published:** 2012-12-06

**Authors:** Jaya K. Rao, Lynda A. Anderson

**Affiliations:** Author Affiliation: Lynda A. Anderson, Centers for Disease Control and Prevention, Atlanta, Georgia.

## Abstract

**Introduction:**

Information on external validity enables public health practitioners to generalize conclusions about an intervention to future or different conditions and is critical to moving research into practice. Prior reviews examining external validity focused on efficacy publications only. Our objective was to determine the extent to which secondary articles could enhance information about external validity presented in efficacy studies.

**Methods:**

We identified a group of interventions recommended by the *Guide to Community Preventive Services* for home-based depression care management for older adults. We searched online databases for secondary articles using a list of the study authors’ names and study acronyms. Five articles were ineligible (intervention was not effective or articles lacked data on external validity) and 14 articles were eligible and reviewed (6 efficacy and 8 secondary articles). We examined 15 elements of external validity based on 4 of the 5 dimensions of the RE-AIM framework: reach, adoption, implementation, and maintenance.

**Results:**

The 6 efficacy studies documented 1 or more elements of reach and implementation, and 5 studies included 1 or more elements of maintenance. Secondary articles included 4 to 9 elements on external validity and addressed 1 to 5 unique elements of external validity not reported in the efficacy publications.

**Conclusion:**

Secondary articles enrich the amount of information about external validity and may be published years before or after the efficacy publication. Reviewing only primary publications of efficacy trials may provide a limited view of external validity, at least for publications describing home-based depression care management.

## Introduction

In 2007, Frederick and colleagues reviewed the evidence for community-based depression interventions for older adults and concluded that sufficient evidence exists to support the effectiveness of depression care management (DCM) strategies (1). Subsequently, the Task Force on Community Preventive Services (2) recommended DCM interventions for the management of depressive disorders among older adults. DCM involves a trained practitioner (or care manager) who works in consultation with primary care providers, psychiatrists, and other members of a multidisciplinary team to provide depression care. This collaborative approach to depression management is based on the chronic care model, which encourages linkages between community and health care systems to provide care that is population-based, evidence-based, and patient-centered (3–5).

To improve the health of the population, we need to focus on translating effective interventions into public health practice. Information on external validity enables public health practitioners to generalize conclusions about a program to future or different conditions and is critical to facilitating research translation (6). Prior reviews (7,8) have documented a dearth of information on external validity in articles describing the primary efficacy results (herein referred to as efficacy articles) of health promotion interventions. No study has examined whether an array of secondary articles contain information about external validity. Thus, our objective was to determine the extent to which secondary articles about DCM interventions enhance information about external validity when combined with information from the efficacy articles identified in the review conducted by Frederick and colleagues. This information can aid practitioners and decision makers in identifying factors important for adoption and implementation of these interventions and making more confident decisions about potentially translating these interventions into practice. We focus on home-based DCM interventions (herein referred to as DCM home interventions) because the evidence shows that these interventions are “applicable to communities in the United States and elsewhere, including diverse home settings (public housing, residential facilities, home care clients)” (2).

## Methods

Frederick and colleagues described their search strategies, search terms, and approaches for identifying eligible interventions for depression management (1). In brief, their systematic review considered all study designs, including interventions that targeted depression as a main or secondary outcome. Eligible studies defined depression on the basis of either clinical criteria (*Diagnostic and Statistical Manual of Mental Disorders*, fourth edition) or a score from an assessment instrument. The authors searched multiple online databases (eg, MEDLINE, PsycInfo, CINAHL) using 12 Medical Subject Headings (eg, aged, depressive disorder, preventive health services) and 21 text words (eg, geriatric, collaborative care, primary care, home). A 14-member expert panel assessed the quality of the studies in different categories of interventions and the magnitude of response or robustness of individual interventions in each category (1). Using an approach similar to that used by the *Guide to Community Preventive Services*, the panel members determined that the DCM home intervention category had adequate data to provide an overall rating, and they rated this category as effective. The DCM home intervention category had 8 efficacy studies.

We excluded 2 efficacy studies (Appendix) from our review (Figure) because the interventions studied had no significant effect on improving depression and were not the focus of subsequent publications demonstrating their efficacy. We compiled a list of all of the authors and study acronyms (eg, PATCH, PEARLS) of the remaining 6 DCM home interventions and used this list to conduct targeted searches of PubMed and Google Scholar. The goal of these searches was to identify all articles, regardless of publication date (ie, even if they predated the efficacy article), that were related to the DCM home interventions and published before June 1, 2012. We identified 11 secondary articles related to 5 DCM home interventions. Three secondary articles did not contain information on external validity and were excluded (Appendix): 2 compared different case finding methods and 1 was a secondary analysis describing patterns of antidepressant use. Thus, 6 efficacy (9–14) and 8 secondary articles (15–22) were eligible for this review. In addition to reviewing efficacy and secondary articles, we also reviewed study websites to determine whether they included information on external validity. We identified these websites by conducting Google searches using study acronyms (eg, PATCH, PEARLS).

**Figure:**
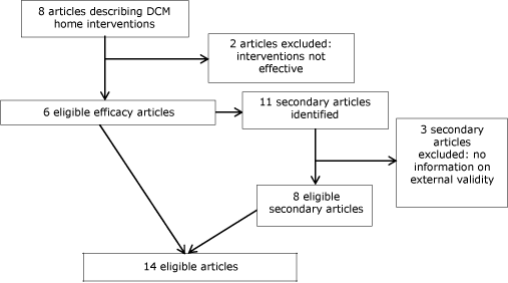
Flow diagram of process used to identify eligible efficacy and secondary articles of depression care management (DCM) home interventions for older adults. The initial 8 articles were identified in the systematic review of depression interventions conducted by Frederick and colleagues (1).

The RE-AIM framework (23) is a program evaluation and planning tool that emphasizes 5 aspects of external validity for facilitating research translation: reach (eg, representativeness of the sample); effectiveness (eg, the intervention’s effect on the primary outcome); adoption (eg, setting and resources necessary to implement intervention); implementation (eg, description of the intervention); and maintenance (eg, program sustainability). Our abstraction tool (Box) was based on this framework and consisted of a checklist (present or absent) of 15 elements of external validity. Because the effectiveness of the 6 DCM home interventions was already documented in Frederick and colleagues’ review and by the *Guide to Community Preventive Services* (2), we collected information on the remaining 4 dimensions of the RE-AIM framework: reach (5 elements); adoption (3 elements); implementation (4 elements); and maintenance (3 elements). We abstracted information on external validity from the efficacy and secondary articles and resolved any discrepancies in abstraction through discussion.

**Box. Checklist of Elements Used to Abstract Information on External Validity From Efficacy Studies and Associated Secondary Articles of Home-Based Depression Care Management Interventions for Older Adults**

**Reach**

- Described a method for identifying the target population
- Described eligibility criteria
- Included participation rate
- Compared participants with nonparticipants
- Compared participants with the broader population

**Adoption**

- Specified necessary skills or expertise for case manager
- Described training procedures for personnel delivering intervention
- Described costs to support intervention personnel

**Implementation**

- Described intervention components
- Described intervention intensity (number and duration of sessions delivered to participants)
- Assessed fidelity to intervention (whether the overall intervention or treatment plans were implemented as planned)
- Described measures of cost per person

**Maintenance**

- Assessed long-term effects (at least 6 months postintervention)
- Described whether intervention program was sustained after the study ended
- Described whether intervention was modified or adapted to another condition

## Results

The 6 eligible efficacy studies of DCM home interventions were published between 1994 and 2004. They were conducted in the United Kingdom (9,14), Australia (12), Canada (10), and the United States (11,13). Five studies were randomized trials, with 1 (10) a pre-post trial. Three studies (9,12,14) enrolled older adults with depression, whereas the other 3 enrolled older adults with minor depression or dysthymia (11), various psychiatric disorders (including depression) (13), and substance abuse with depression (10).

One efficacy study (11) reported information on 9 of 15 elements of external validity, with the remainder reporting 6 to 8 elements (Table). The 6 studies included information on at least 1 element of reach and implementation, and 5 of the 6 studies included information on at least 1 element of maintenance. All 6 studies included information on 3 elements of reach (target population, eligibility criteria, and participation rate) and described the intervention components. Four studies described the specific level of training (eg, psychiatric nurse) of the care manager (10,11,13,14) and included information on the number and duration of intervention sessions delivered to participants (ie, intervention intensity) (9,11,13,14). Two studies (9,12) described whether the overall intervention or treatment plans were implemented as planned (ie, intervention fidelity) and only 1 study (11) reported the intervention costs per subject. Five of the 6 described the long-term effects (6 months or greater) of the intervention on depression outcomes (9–13). No study presented information on comparisons of the participants with nonparticipants or the target population, funding requirements for intervention personnel, whether the program was sustained after the end of the study, or any necessary modifications or adaptations that were made to the intervention for another condition.

**Table:** Elements of External Validity^a^ Presented in Efficacy and Secondary Articles of Home-Based Depression Care Management Interventions^b^ for Older Adults

Elements	Banerjee (Efficacy) (9)	Brymer (Efficacy) (10)	Ciechanowski (11)		Llewellyn-Jones (12)		Rabins (13)		Waterreus (14)	
			Efficacy	Secondary	Efficacy	Secondary	Efficacy	Secondary	Efficacy	Secondary
**Reach**										
Method to identify target population	X	X	X		X		X	(18)	X	(15)
Eligibility criteria	X	X	X		X		X		X	(15)
Participation rate	X	X	X		X		X		X	(15)
Participants to nonparticipants comparisons										
Participants to target population comparisons										
**Adoption**										
Level of expertise (eg, training) of care manager		X	X			(19)	X	(18)	X	(15)
Training procedures for intervention personnel			X			(19)	X	(18)		
Funding of personnel						(19)				
**Implementation**										
Intervention components	X	X	X	(20)	X	(19)	X	(18)	X	(15,16)
Intervention intensity (number and duration of sessions delivered to participants)	X		X				X	(18)	X	(15–17)
Fidelity to intervention (whether the overall intervention or treatment plans were implemented as planned)	X				X					(15–17)
Intervention costs per person			X							
**Maintenance**										
Long-term assessment (time)	X (6 months)	X (6 months)	X (12 months)		X (9.5 months)		X (26 months)	(18) (9 years)		(17) (6-23 months)
Program sustained after end of study				Web^c^		(19)		(18)		
Intervention modified or adapted to another condition				(21,22)		(19)				

a The external validity elements were based on the RE-AIM framework (23). Elements reported in the efficacy article are denoted by an “X,” and elements present in the secondary articles are denoted by the reference number.

b Home-based depression care management interventions were identified in the systematic review of depression interventions conducted by Frederick and colleagues (1).

c The authors posted information on program sustainability on a study website (www.pearlsprogram.org).

One study (10) had no related secondary articles, 1 (9) had no eligible secondary articles (Appendix), 2 (12,^13^) had 1 eligible secondary article apiece, and 2 (11,14) had multiple eligible secondary articles. The 8 eligible secondary articles were published between 1992 and 2011 and were related to 4 efficacy studies. Waterreus presented details about the study methods in a secondary article (15) that predated publication of the efficacy article. Another study (11) had 2 secondary articles published 6 and 7 years after publication of the efficacy findings.

Overall, secondary articles included information on 4 to 9 elements of external validity. For 2 (13,14) of the 4 efficacy studies, secondary articles addressed 1 or more elements of reach, adoption, implementation, and maintenance. Three secondary articles (15,18,19) described aspects of adoption, such as required skills or expertise of the care managers and procedures for training personnel. A secondary paper (19) was the only paper among all of the articles reviewed (efficacy and secondary) to describe the funding required to support intervention personnel. Secondary articles to studies by Rabins and Waterreus provided more details about intervention intensity (15–18) and fidelity to the intervention treatment plan (15–17). Finally, secondary articles to the 4 efficacy studies that had related secondary articles provided information either on long-term effects of the intervention on depression (17,18), program sustainability (18,19), or modifications (18,19). Authors of 1 efficacy study (11) posted information on program sustainability on a study website (www.pearlsprogram.org) and published 2 articles (21,22) on the effects of adaptation of the intervention to people with a specific chronic disease (epilepsy).

Secondary publications addressed 1 to 5 unique elements of external validity not presented in the efficacy publications. Considering the external validity data presented in the efficacy and secondary articles together, Ciechanowski (11) and Llewellyn-Jones (12) addressed the most elements (11 each). Overall, Rabins (13) addressed 9 elements of external validity and Waterreus (14) addressed 8 elements. Efficacy articles by Banerjee (9) and Brymer (10), which did not have secondary publications, addressed 7 and 6 external validity elements, respectively.

Most studies presented information on reach and implementation. Reach was described either in the efficacy or secondary articles, focusing mainly on descriptions of the target population, eligibility criteria, and participation rate. No study provided information necessary to assess whether the participants were representative of the target population or how comparable they were to nonparticipants. In terms of adoption, only 1 efficacy study (12) provided information on funding requirements for intervention personnel in a secondary paper (19). Regarding implementation, all of the efficacy articles described the intervention, but 2 did not provide information on intervention intensity. Only 1 efficacy study (11) provided information on intervention costs, and only 2 efficacy articles (9,12) and 3 secondary articles (15–17) described the fidelity of intervention delivery to the original intervention or treatment plans. Five efficacy studies (9–13) reported on maintenance, and for 1 efficacy study (14), this information was only reported in the secondary article (17). Two efficacy studies (11,12) presented information on program modifications or adaptations in secondary articles (19,21,22). Four studies (11–14) provided information on lessons learned in the efficacy or secondary articles (14,18,19) or on a website (www.pearlsprogram.org).

## Discussion

During the past several years, policy makers and investigators have noted the need for more rapid translation of advances in prevention and treatment into clinical and public health practice (24–26). At the same time, there is growing recognition by the public health community that promising interventions, which are often developed under ideal circumstances, may be difficult to replicate in everyday practice (24,27). This failure of the “research to practice” transition may be due to a lack of critical information on the contextual factors of external validity that decision makers need to determine whether a particular intervention is applicable to their specific settings, populations, and available resources (5,24). Our findings indicate that secondary articles contain valuable information on external validity.

Like prior reviews of various public health interventions (7,8), our review of the external validity of DCM home interventions found that all contained information on aspects of reach, such as target population, eligibility criteria, and participation rate. The high degree of reporting on reach is understandable, as these details provide information on the representativeness of the subjects receiving the intervention and is the 1 aspect of external validity emphasized in reporting guidelines such as the CONSORT criteria. We also found that the efficacy articles contained little information related to elements of adoption, implementation, and maintenance. However, we encourage caution in directly comparing our findings to other reviews due to differences in how researchers may have operationalized external validity for the purpose of abstracting information from studies.

Our review extends the methods used in other reviews of external validity because we also reviewed all secondary articles related to the efficacy studies describing DCM home interventions. Because of this novel approach, we found that secondary articles enhanced the information on external validity (eg, implementation) that was presented in the efficacy articles. However, we also found that the secondary articles addressed 1 to 5 unique elements of external validity that were not addressed in the efficacy publications. In particular, secondary articles were instrumental in describing factors related to maintenance, such as program sustainability and modification and adaptation of the interventions to other conditions. None of this valuable information was available in the efficacy articles. Thus, by reviewing secondary articles, some of which were published before or years after the efficacy article, we gained a more complete understanding of the external validity of DCM home interventions, which may facilitate the uptake of these interventions by the public health community.

The lack of information about external validity in efficacy articles may relate to reporting guidelines that have been widely adopted by journals and place emphasis on reporting elements related to the internal validity of interventions (24,28). In 2006, 12 editors of public health and health promotion journals recommended that authors provide more information on external validity (6). In particular, they called for more frequent reporting of the following aspects of external validity:

- Recruitment and selection procedures, participation rates, and representativeness of participants, intervention staff, and delivery settings.
- Level and consistency of implementation among program components, settings, staff, and time.
- Intervention’s effect on various outcomes, especially outcomes important to populations, practitioners, and decision makers (eg, quality of life, program costs, adverse consequences).
- For follow-up reports, information on the rate of attrition at all levels (ie, study participants, intervention staff, and delivery settings), long-term effects on outcomes, and program institutionalization, modification, or termination.

We agree with these recommendations and suggest that secondary articles may represent another venue in which investigators can provide more details regarding external validity. We propose that practitioners interested in a particular intervention perform a thorough search of the literature to identify associated articles that may contain this information. Moreover, summaries of external validity information that are posted on a program website and that contain links to publications would help to ensure greater accessibility by the practice community.

Several strengths and limitations should be considered when reviewing our findings and their implications. A strength of this review is our use of elements from a well-established framework to abstract information on external validity from the articles. Second, by limiting the focus of our review to efficacy studies of effective DCM home interventions, our approach more likely reflects the real-world approach of decision makers, because they are less likely to consider implementing interventions that are not effective. Our review of secondary articles shows that they can contribute toward a more comprehensive understanding of the external validity of an intervention.

Conversely, our findings are based on information abstracted from publications only; we made no attempt to contact study investigators. Thus, we do not know why some external validity elements were reported and others were not. However, our approach is likely to replicate the typical approach of practitioners when they examine an intervention, and our findings reinforce the need to expand beyond a review of efficacy publications. Second, our review focused on the external validity of DCM home interventions identified in a 2007 systematic review of depression interventions (1), and efficacy articles describing new DCM home interventions may have been published since then. Our primary objective was to complement our understanding of the internal validity of effective DCM home interventions identified by Frederick and colleagues and recommended by the *Guide to Community Preventive Services* by examining the external validity of these interventions. Thus, identifying and characterizing the internal validity of new DCM home interventions was beyond the scope of our study. However, in our searches for secondary articles, we did update the findings related to the interventions identified in the 2007 review, including the adaptation of the PEARLS program to adults with a specific chronic condition, epilepsy (21,22).

Our findings indicate that external validity data may be present in articles other than efficacy publications. Practitioners who are interested in a particular intervention should consider reviewing secondary articles, because these articles may contain additional information about external validity that can aid in research translation. These articles may be published years before or after the efficacy publication. Investigators could facilitate a more rapid translation of research into practice by summarizing and posting key aspects of external validity on accessible websites and providing links to relevant publications as they become available.

## Appendix. List of Efficacy and Secondary Articles That Were Excluded From This Review

**Efficacy articles of depression care management interventions (home)**

Reason for exclusion: interventions were not effective in improving depression outcomes. These 2 interventions were included in the systematic review of depression interventions conducted by Frederick and colleagues ( Frederick JT, Steinman LE, Prohaska T, Satariano WA, Bruce M, Bryant L, et al. Community-based treatment of late life depression: an expert panel-informed literature review. Am J Prev Med 2007;33[3]:222–49).

1. Arthur AJ, Jagger C, Lindesay J, Matthews RJ. Evaluating a mental health assessment for older people with depressive symptoms in general practice: a randomised controlled trial. Br J Gen Pract 2002;52(476):202–7.

2. Flaherty JH, McBride M, Marzouk S, Miller DK, Chien N, Hanchett M, et al. Decreasing hospitalization rates for older home care patients with symptoms of depression. J Am Geriatr Soc 1998;46(1):31–8.

**Secondary articles**

Reason for exclusion: compared case finding instruments and contained no information on external validity.

1. Banerjee S, Shamash K, MacDonald AJ, Mann AH. The use of the SelfCARE(D) as a screening tool for depression in the clients of local authority home care services — a preliminary study. Int J Geriatr Psychiatry 1998;13(10):695–9.

2. Blanchard MR, Waterreus A, Mann AH. The nature of depression among older people in inner London, and the contact with primary care. Br J Psychiatry 1994;164(3):396–402.

Reason for exclusion: described secondary analysis and contained no information on external validity.

1. Lakey SL, Gray SL, Ciechanowski P, Schwartz S, LoGerfo J. Antidepressant use in nonmajor depression: secondary analysis of a Program to Encourage Active, Rewarding Lives for Seniors (PEARLS), a randomized controlled trial of older adults from 2000 to 2003. Am J Geriatr Pharmacother 2008;6(1):12–20.
